# *Listeria* meningitis diagnosed by blood culture with fever, neurological symptoms, and no meningeal irritation signs

**DOI:** 10.1002/ccr3.8020

**Published:** 2023-10-10

**Authors:** Masaki Tago, Risa Hirata, Yuka Hirakawa, Seijiro Makio, Toru Oishi, Masahiko Nakamura, Shun Yamashita, Yoshinori Tokushima, Midori Tokushima, Naoko E. Katsuki, Hidetoshi Aihara, Motoshi Fujiwara

**Affiliations:** ^1^ Department of General Medicine Saga University Hospital Saga Japan

**Keywords:** aged 80 and over, blood culture, meningitis *Listeria*, neurologic manifestations

## Abstract

**Key Clinical Message:**

*Listeria* can cause neurological symptoms in immunocompromised and older patients. Additionally, it is impossible to rule out meningitis by the absence of typical meningeal irritation signs. Therefore, patients with fever and neurological impairments should be rapidly examined for blood and cerebrospinal fluid cultures to rule out *Listeria* meningitis.

**Abstract:**

A woman in her 90s developed fever, dysarthria, and transient disturbance of consciousness. Physical examination revealed no meningeal irritation signs. *Listeria monocytogenes* were detected in her blood culture the following day. Because of an increased number of cells in cerebrospinal fluid, she was diagnosed with *Listeria* meningitis.

## 1 INTRODUCTION

Fever can be caused by infectious diseases, noninfectious inflammatory diseases, and malignancies, of which infectious diseases account for more than half of all hospitalizations.^1^ Among infectious diseases, respiratory and urinary tract infections are the most common.^1^ Although meningitis is not a frequent cause of fever, meningitis due to various causes (including bacteria) should be considered during differential diagnosis of infections of unknown origin.^1^, ^2^ Typical symptoms of bacterial meningitis are headache, fever, neck stiffness, and altered mental status, which are found in most cases.^3^ In addition, typical physical findings are neck stiffness, Jolt accentuation, Kernig's sign, and Brudzinski's sign.^4^ In clinical practice, meningitis is commonly suspected when a patient develops meningeal irritation symptoms such as neck stiffness and Jolt accentuation in addition to fever and headache. However, these signs and findings are not inevitable, and meningitis cannot be ruled out by their absence. Furthermore, older patients needing emergency care often exhibit disturbed consciousness with delirium accounting for 8%–10% of cases and stupor and coma for 5%–9%.^5^ Therefore, patients with fever and disturbance of consciousness should be carefully evaluated.

*Streptococcus pneumoniae*, *Neisseria meningitidis*, and *Listeria monocytogenes* are the most common causes of community‐acquired bacterial meningitis (72%, 11%, and 5% of cases, respectively).^3^ Because immunocompromised patients with malignancies and older populations are more easily infected with *Listeria*,^6^ care should be taken to correctly diagnose bacterial meningitis in older patients. Additionally, *Listeria* meningitis is less likely to show the typical findings of common bacterial meningitis, such as a low cell count in cerebrospinal fluid, and it can be difficult to detect *Listeria* by Gram staining.^7^, ^8^ In some cases, it is necessary to wait for the results of blood and cerebrospinal fluid cultures,^7^, ^8^ which makes it difficult to diagnose in the early period.

We herein report a case of *Listeria* meningitis in an older patient who presented with fever, dysarthria, transient disturbance of consciousness, and no symptoms of meningeal irritation.

## 2 CASE REPORT

A woman in her 90s, healthy enough to do farm work, had a disturbance of consciousness, lack of eye contact, back pain, and dysarthria in the morning on Day 0. She was transferred to the emergency room. She had no history of alcohol consumption, falls, or trauma. She had a history of paroxysmal atrial fibrillation, hypertension, osteoporosis, and chronic heart failure and had been taking eldecalcitol (0.75 μg), raloxifene (60 mg), valsartan (80 mg), amlodipine (5 mg), carvedilol (2.5 mg), and apixaban (2.5 mg). On the way to the emergency room, her consciousness and dysarthria improved. Physical examination on arrival at the hospital revealed a Glasgow Coma Scale of E4V4M6, body temperature of 39.4°C, blood pressure of 163/88 mmHg, heart rate of 94 beats/min with a regular rhythm, respiration rate of 23 breaths/min, and 94% saturation of percutaneous oxygen in ambient air. She had left costovertebral angle knocking pain but no meningeal irritation signs such as Jolt accentuation, neck stiffness, and Kernig's sign. She showed no abnormalities in breath, heart sounds, or abdomen. A neurological physical examination revealed mild dysarthria, with normal muscle strength and tendon reflexes in the extremities and no abnormal reflexes. Electrocardiogram showed a sinus rhythm without arrhythmias, including atrial fibrillation. The results of the laboratory examination are provided in Table 1. Venous blood gas analysis showed a pH of 7.445, pCO_2_ 40.9 mmHg, HCO_3_^−^ 27.7 mM/L, and lactate 2.6 mmol/L. Urinalysis was negative for white blood cells and positive for nitrite. Computed tomography of the brain, chest, and abdomen (without contrast enhancement) and magnetic resonance imaging of the brain showed no abnormalities.

**TABLE 1:** Laboratory findings on admission.

Complete blood count			Chemistry profile			Na	139	mmol/L
White blood cells	11.2	10^9^/L	TP	7.0	g/dL	K	2.9	mmol/L
Neutrophil	80.6	%	Albumin	3.6	g/dL	Cl	99	mmol/L
Lymphocyte	14.3	%	BUN	5.39	mmol/L	Ca	2.14	mmol/L
Red blood cells	3.61	10^12^/L	Cr	75.0	μmol/L	CRP	20.5	mg/L
Hemoglobin	123	g/L	eGFR	53.6	mL/min/1.73	Urine		
Hematocrit	35	%	T‐Bil	0.014	mmol/L	Blood	−	
MCV	97.0	fl	Glucose	8.43	mmol/L	Leukocytes	−	
Platelets	167	10^9^/L	Ammonia	14.6	μmol/L	pH	5	
Coagulation profile			AST	467	nkat/L	Glucose	+	
PT‐INR	1.26		ALT	217	nkat/L	Total proteins	2+	
APTT	29.5	sec	LDH	4300	nkat/L	Bilirubin	+	
Fibrinogen	4.85	g/L	ALP	1317	nkat/L	Ketones	−	
FDP	3.5	μg/mL	γ‐GTP	250	nkat/L	Specific gravity	1.025	
D‐dimer	1030	μg/L	CK	700	nalt/L	Nitrite	+	

Abbreviations: ALP, alkaline phosphatase; ALT, alanine aminotransferase; APTT, activated partial thromboplastin time; AST, aspartate aminotransferase; BUN, blood urea nitrogen; Ca, calcium; CK, creatine phosphokinase; Cl, chloride; Cr, creatinine; CRP, C‐reactive protein; eGFR, estimated glomerular filtration rate; FDP, fibrin degradation products; K, potassium; LDH, lactate dehydrogenase; MCV, mean corpuscular volume; Na, sodium; PT‐INR, prothrombin time‐international normalized ratio; T‐Bil, total bilirubin; TP, total protein; γ‐GTP, γ‐glutamyl transpeptidase.

A urinary tract infection was suspected, and she was hospitalized and started on intravenous ceftriaxone (2 g/day). However, blood cultures collected on admission showed gram‐positive rods on Day 1. Therefore, antibiotic therapy was changed to intravenous ampicillin/sulbactam (9 g/day). On Day 2, *Listeria monocytogenes* were identified as the causative bacteria. Based on the symptoms of fever, disturbed consciousness, and dysrhythmia, we strongly suspected *Listeria* meningitis and performed a cerebrospinal fluid examination. This showed an initial pressure of 10 cm H_2_O with clear, pale yellow cerebrospinal fluid (116 g/L protein and 2.8 mmol/L glucose [blood glucose was 11.4 mmol/L]). White blood cell counts were 604 cells/mm^3^ with 68.9% mononuclear cells and 31.1% polynuclear cells. The cerebrospinal fluid culture was negative after administration of ceftriaxone, which is ineffective against *Listeria* monocytogenes. Therefore, the cerebrospinal fluid findings were atypical but consistent with *Listeria* meningitis. Consequently, she was diagnosed with invasive *Listeria* infection, including bacteremia and meningitis, and treated with intravenous ampicillin (8 g/day) for 15 days.

After admission, she did not develop any further disturbance in consciousness, dysarthria, or any other neurological abnormality. However, her activity of daily living worsened with hospitalization. She was transferred to another hospital on Day 16 for continuous antibiotic treatment and rehabilitation. She was discharged to home on Day 83.

## 3 DISCUSSION

The present case was an older patient without any meningeal irritation signs. Blood cultures led to a diagnosis of *Listeria* meningitis. She developed fever, transient disturbance of consciousness, and dysarthria. Bacterial meningitis is reported to present with headache (83%), fever >38°C (74%), neck stiffness (74%), nausea (62%), rash (8%), cranial nerve palsy (9%), and aphasia or partial paralysis (22%).^3^ Only 41% of patients with bacterial meningitis present with all the classical triad of fever, neck stiffness, and disturbance of consciousness.^3^ Meningeal irritation signs include neck stiffness, Jolt accentuation, Kernig's sign, and Brudzinski's sign (with sensitivities and specificities in meningitis being 46.1% and 71.3%, 52.4% and 71.1%, 22.9% and 91.2%, and 27.5% and 88.8%, respectively).^9^ Hence, the absence of the classical triad or meningeal irritation signs cannot rule out bacterial meningitis. In contrast, *Listeria* meningitis typically presents with fever (91%), headache (67%), and vomiting (46%). Moreover, *Listeria* meningitis is characterized by cerebellar dysfunction and a more focal neurological impairment than bacterial meningitis caused by other bacteria (32% vs. 12% and 12% vs. 2%, respectively).^7^ Because the symptoms of *Listeria* meningitis are different from those common forms of bacterial meningitis, it is necessary to consider *Listeria* meningitis when a patient presents with unexplained fever and neurological impairments, such as dysarthria and disturbed consciousness, even if they do not have the typical symptoms and signs of bacterial meningitis.

When bacterial meningitis is suspected, it is necessary to quickly perform a blood culture and cerebrospinal fluid examination. In the present case, *Listeria* meningitis was suspected because the blood cultures obtained before administration of antibiotics showed a positive result for *Listeria monocytogenes*. With bacterial meningitis, blood cultures show a positive result in 30% of cases, Gram staining of cerebrospinal fluid in 24.5%, and cerebrospinal fluid cultures in 91.3%.^10^ In contrast, studies of *Listeria* meningitis have found positive blood cultures in 46%–63% of cases, positive Gram staining of cerebrospinal fluid in 28%–33%, and positive cerebrospinal fluid cultures in 93%.^7^, ^8^ This suggests that *Listeria* meningitis may be more readily detected in blood cultures than other types of bacterial meningitis. *Listeria* is the third most common cause of bacterial meningitis.^3^ Gram staining of cerebrospinal fluid can be negative in bacterial meningitis treated with antibiotics prior to cerebrospinal fluid examination. Consequently, *Listeria* can be mistaken for Streptococcus Pneumoniae or be difficult to stain^11^, ^12^; therefore, misdiagnosis is possible with only a cerebrospinal fluid culture. Blood cultures are therefore required to correctly diagnose *Listeria* meningitis. In addition, the median leukocyte count in cerebrospinal fluid of patients with *Listeria* meningitis is reportedly 550 ± 2480 cells/mm^3^ with a median ratio of glucose in cerebrospinal fluid to blood of 0.26 ± 0.24. Both values are lower than with other types of bacterial meningitis,^7^ and are similar to the findings in the present case. Because the cephem antibiotics used in empirical therapy of broad‐spectrum antibiotics for bacterial meningitis are ineffective for *Listeria* meningitis,^8^ penicillin G, ampicillin, or amoxicillin are commonly used, sometimes in combination with aminoglycoside antibiotics.^13^ Recognizing the characteristic cerebrospinal fluid findings of *Listeria* meningitis may lead to appropriate diagnosis and treatment.

## 4 CONCLUSION

Bacterial meningitis can present with an absence of meningeal irritation signs, and *Listeria* meningitis is more likely to present with neurological symptoms than is typical with bacterial meningitis. When a patient develops unexplained fever with neurological impairments, even without meningeal irritation signs, physicians should consider bacterial meningitis, especially *Listeria* meningitis, and quickly perform a blood culture in addition to a cerebrospinal fluid examination.

## AUTHOR CONTRIBUTIONS

**Masaki Tago:** Conceptualization; funding acquisition; project administration; supervision; writing – original draft; writing – review and editing. **Risa Hirata:** Conceptualization; methodology; writing – original draft. **Yuka Hirakawa:** Conceptualization; writing – review and editing. **Seijiro Makio:** Conceptualization; writing – review and editing. **Toru Oishi:** Conceptualization; writing – review and editing. **Masahiko Nakamura:** Conceptualization; writing – review and editing. **Shun Yamashita:** Conceptualization; writing – review and editing. **Yoshinori Tokushima:** Conceptualization; writing – review and editing. **Midori Tokushima:** Conceptualization; writing – review and editing. **Naoko E. Katsuki:** Conceptualization; writing – review and editing. **Hidetoshi Aihara:** Conceptualization; writing – review and editing. **Motoshi Fujiwara:** Conceptualization; writing – review and editing.

## FUNDING INFORMATION

There is no funding for this article.

## CONFLICT OF INTEREST STATEMENT

The authors declare no conflicts of interest associated with this manuscript.

## ETHICS STATEMENT

This manuscript conforms to the provisions of the Declaration of Helsinki in 1995 (as revised in Brazil 2013).

## CONSENT

Written informed consent was obtained from the patient to publish this report in accordance with the journal's patient consent policy.

## Data Availability

The data that support the findings of this study are available from the corresponding author upon reasonable request.
